# Challenges of COVID-19 Vaccination Delivery in Pakistan

**DOI:** 10.12669/pjms.37.7.4489

**Published:** 2021

**Authors:** Shahan Tariq, Muhammad Ammar Bin Hamid

**Affiliations:** 1 Shahan Tariq, Combined Military Hospital, Rawalpindi, Pakistan; 2 Muhammad Ammar Bin Hamid, Combined Military Hospital, Rawalpindi, Pakistan

In the 21^st^ century, the world has turned into a global village by the means of easy access where traveling capabilities have reached beyond imagination. However, the downside to this has just come to light with the spreading of coronavirus causing havoc, wave after wave. Industries, healthcare setups, large firms, airlines, etc all the way down to small businesses have taken a huge toll due to the COVID-19 pandemic. To control spread, governments across the world have had to call for desperate measures including complete lockdowns of ever-bustling mega-cities. Such a course of action is not sustainable in the long-term, though. Vaccination may provide us with a feasible solution that is more economical as well as practical and perhaps limit viral transmission.

In Pakistan lack of government funds, fewer healthcare setups to cater to the masses, hospitals working to their full capacity, high illiteracy rates, pollution along with poor hygiene habits, and over-crowding are a number of factors not only contributing to the disease spread but potentiating it as well.^1^ To counter these factors, no less than a cumulative role ranging from government authorities to individuals shall be enough. The most integral role is currently being played by the frontline healthcare workers who have been actively involved in the management, education, and counseling at the grass-root level. Therefore, they shall be instrumental in coping with the tremendous challenge of nationwide delivery of vaccination as well as the removal of any hesitancy towards it.

The Expanded Program on Immunization (EPI) has been assigned by the government to supervise the administration of vaccinations across the country. At present, EPI concentrates on immunization of the pediatric population with regard to eight preventable illnesses allowing the employees to be in touch with the local populace all around the year.^2^ This association can be harnessed for the education of the masses regarding SARS-CoV-2 and its vaccination with the providence of accurate knowledge and correction of any false information or misleading claims.

It would be an injustice to leave out the contribution of Lady Health Workers (LHWs) in Pakistan. LHWs constitute 90,000 plus individuals, who provide coverage with respect to all aspects of maternal and newborn/child healthcare to approximately 60 percent of the Pakistani population, mostly residing in rural areas.^3^ With the passage of time, they have developed into a vital infrastructure linking the basic health units and first-line healthcare setups directly to the communities. They also provide a very cost-effective solution and can have a significant impact in the present situation, where we are faced with the challenge of COVID-19 vaccination on a country-wide scale. Due to the level of success they have achieved and the trust they have built in society, they can play an extremely important role in enlightening the general population regarding the COVID-19 vaccine as well as for the removal of any vaccination hesitancy.

Our media has played a vital role in raising awareness regarding COVID-19 and the subsequent dissemination of information to the general public.^4^ Various platforms such as news channels, advertisements, talk shows, and even phone companies have been actively involved in conveying timely and accurate information. The most pragmatic approach would be to limit the distribution of advice regarding this disease as well as upcoming vaccination via appropriate channels involving scientists, researchers, and health care professionals. All of these measures will eventually help in the timely and prompt delivery of COVID-19 vaccination all over Pakistan.
